# Barriers to health in women of reproductive age living with or at risk of non-communicable diseases in Nigeria: a Photovoice study

**DOI:** 10.1186/s12905-022-02146-6

**Published:** 2023-01-02

**Authors:** Imo Etuk, Amira Iwuala, Kendra Njoku, Bosoye Olagbegi, Ayoposi Ogboye, Jonas Kofi Akpakli, Ugo Okoli, Kathleen Hill, Oniyire Adetiloye, Donald Imosemi, Victoria Omoera, Folashade Oludara, Iniobong Ekong, Olubunmi Alabi, Nneka Mobisson

**Affiliations:** 1 mDoc Healthcare, 1a Hakeem Dickson Drive, Off T.F. Kuboye Street, Lekki Phase 1, Lagos, Nigeria; 2 Jhpiego, Abuja, Nigeria; 3 Lagos State Ministry of Health, Lagos, Nigeria; 4 Federal Capital Territory Health & Human Services Secretariat, Abuja, Nigeria

**Keywords:** Non-communicable disease, Photovoice, Women of reproductive age, Maternal mortality, Barriers, Healthcare

## Abstract

**Background:**

Nigeria has one of the highest maternal mortality ratios (MMR) globally with an MMR of 512 (per 100,000 live births) and the proportion of maternal deaths due to non-communicable diseases (NCDs) is increasing. While evidence shows that many of these deaths are preventable, limited attention is being paid to the unique vulnerabilities and experiences of women of reproductive age (WRA) with NCDs and their risk factors, as well as the barriers to the screening, diagnosis, and management of these diseases in Nigeria.

**Methods:**

This study explored the lives of WRA in Lagos and Federal Capital Territory in Nigeria from May to June 2019 using a community-based participatory research (CBPR) methodology called Photovoice which is aligned with CBPR as it includes procedures such as the identification of important community issues, discussion of photo assignments and data analysis. Twenty-four women of reproductive age were provided with digital cameras and trained on how to capture photos that conveyed their current health, healthcare utilization and engagement, and experience journeys. Individual interviews with the women were held for an in-depth exploration of the photographs. The data was then analysed thematically.

**Results:**

Six distinct themes were identified across the barriers highlighted by the women: *food and nutrition, home and family, neighborhood-built environment, economic instability, religion and spirituality and low prioritization of self-care.* These themes captured the challenge of reduced agency, limited contribution and participation, and a complex relationship between visible and invisible illness.

**Conclusion:**

The perspectives of WRA in Nigeria obtained through this qualitative research provided a strong substratum for understanding the environmental barriers that predispose WRA to NCDs in Nigeria. The results of the study are useful for the improvement of woman-centred services of prevention, diagnosis, and management of NCD risk factors across the maternal and reproductive health care continuum in Nigeria.

## Introduction

Nearly 20% of global maternal deaths occur in Nigeria [1]. Approximately 600, 000 maternal deaths occurred in the country between 2005 and 2015 [1]. A woman living in Nigeria has a 1 in 22 lifetime risk of dying during pregnancy, childbirth or postpartum/post-abortion, whereas the risk in most high-income countries is only 1 in 4900 [1].

On a global level, the number of maternal deaths due to direct obstetric causes is declining but the proportion of indirect maternal deaths (i.e., deaths due to a previously existing disease, or disease that developed during pregnancy that was not due to direct obstetric causes but was aggravated by physiological effects of pregnancy) is rising from less than 10% to almost 11% [2]. Nigeria is experiencing this obstetric transition with an increasing proportion of indirect causes due to NCDs being the root causes of maternal mortality [2, 3]. For instance, Hypertension Pregnancy Disorders (HPDs) in women are a leading cause of maternal death in Nigeria, [3, 4]. While prevalence studies on maternal NCDs are sparse, prevalence is up to 13.9% in the urban setting in Nigeria [5]. Both gestational Diabetes Mellitus (GDM) and chronic hypertension are risk factors for one of the main drivers of maternal mortality in Nigeria: Pre-Eclampsia/Eclampsia [6].

Nigeria and other sub-Saharan African countries are undergoing an epidemiologic transition with a decline in the prevalence of communicable diseases and a steady increase in the incidence of NCDs being a driver of premature death across the population [7]. In fact, by 2030, NCDs are projected to contribute more to mortality and morbidity on the continent than communicable and infectious diseases [8]. Many countries, including Nigeria, are experiencing a rise in NCD prevalence among younger age groups due to predisposing factors such as physical inactivity, poor eating habits, and sedentary lifestyles [2, 7].

It is paramount for women of reproductive age to receive reliable quality care to prevent and manage chronic conditions before, during and after pregnancy to decrease the risk of maternal morbidity and mortality [9]. Hence, understanding the environmental realities that women living with or at risk for NCDs are exposed to, can highlight opportunities and interventions to improve maternal health in low- and middle-income countries. However, although adverse outcomes in pregnancy can be reduced with early screening and dietary and lifestyle modifications to prevent and manage NCDs, there is a dearth of data on the realities and enabling elements that shape the health status of WRA living with or at risk for NCDs such as diabetes, hypertension [2].

Previous studies have assessed indirect and direct causes of maternal mortality in Nigeria, using mainly conventional qualitative and quantitative research methodologies [9–11]. Unlike these studies, Photovoice enables women to document and communicate the barriers they face themselves. Photovoice can also increase community engagement and foster the self-efficacy of participants [12]. This PhotovoicePhotovoice study aims to augment the limited literature on the risk and enabling factors that WRA experience that affect their perceptions of health, particularly NCDs, and management practice in Nigeria. In this study, we aimed to: (1) explore and document women’s needs, experience of care, and preferences for reproductive, maternal, and NCD services; (2) identify contributing barriers to prevention, control, and management of NCDs that WRA encounter from the perspective of women living with or at risk of these chronic diseases; and (3) generate opportunities for Nigerian political and community interventions and prioritization that address the multifaceted socio-economic implications in the risk factors for NCDs in order to mitigate maternal mortality in the country.

## Methods

### Study design

This was a descriptive cross-sectional study conducted between May and June 2019 which made use of Photovoice, a community-based participatory research method to gain deeper insights into the barriers that women living with or at risk of chronic diseases face and to understand how factors leading to the indirect causes of maternal mortality can be addressed in Nigeria. Photovoice explores contextual elements—such as the built and social environment—to reveal health barriers and opportunities in the community [13, 14]. This study explores the different obstacles Nigerian women navigate that hinder the management of their chronic illnesses.

This PhotovoicePhotovoice study was part of the Reducing Indirect Causes of Maternal Mortality and Morbidity (RICOM3) project which aims to reduce the incidence of maternal mortality and morbidity due to indirect causes associated with preeclampsia and eclampsia via an integrated quality of care model approach. Another part of the RICOM3 project is a larger formative research study that assessed the prevalence of NCDs and risk factors of 639 WRA in FCT and Lagos, Nigeria.

### Study area, setting, and selection of participants

**Fig. 1:**
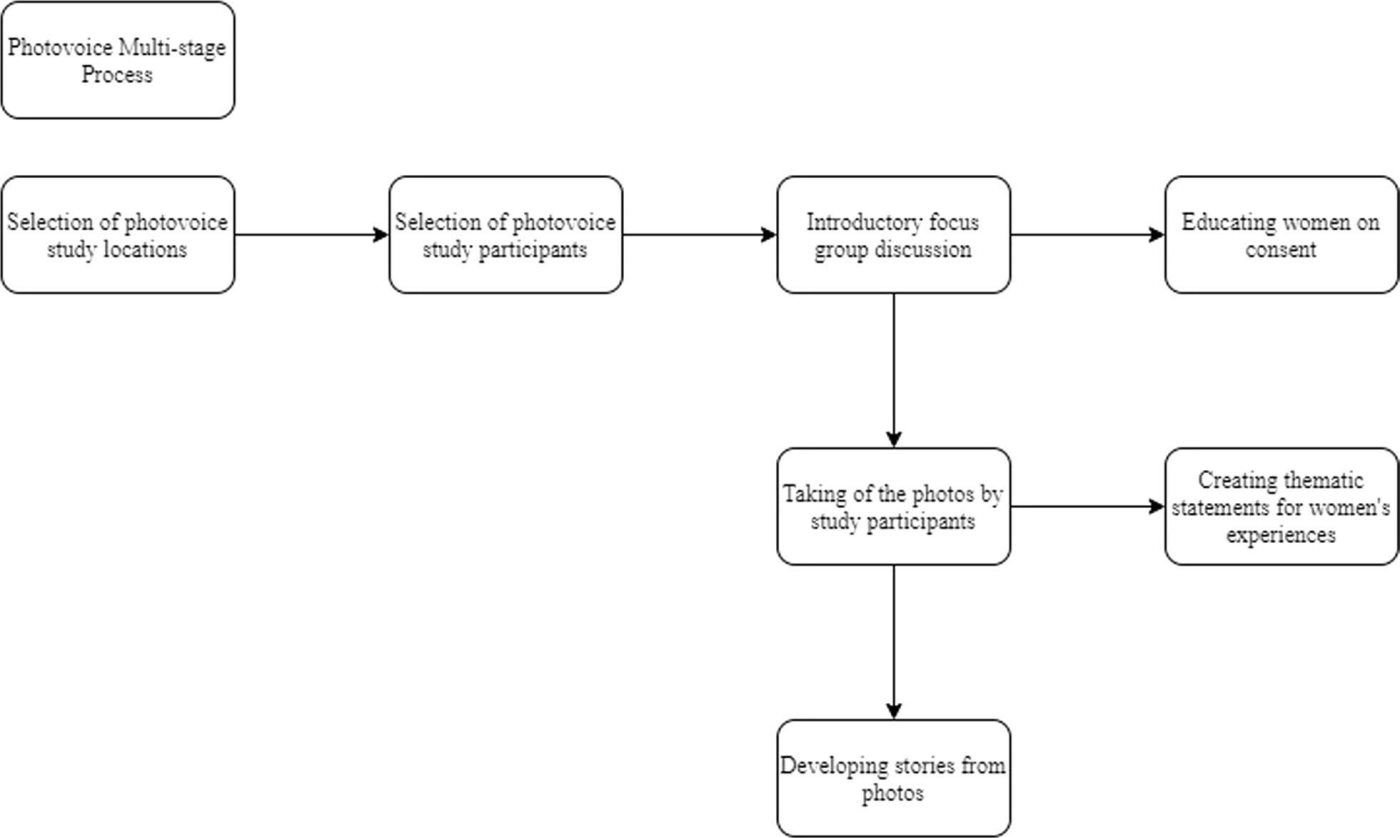
The multi-stage process of the PhotovoicePhotovoice study

We utilized a multi-staged process to conduct the PhotovoicePhotovoice study (Fig. 1).

#### Phase 1: selection of Photovoice study locations

The PhotovoicePhotovoice study was conducted in four local government areas (LGAs) across Lagos and FCT: two of Nigeria’s most populous and diverse states. Both states were selected based on their high burden of maternal mortality and given their challenges with health infrastructure, human resources for health, coordination of care and poor health insurance coverage. Two of the most populous LGAs in each state were selected, intentionally covering rural/peri-urban, and urban communities in the states: Ikorodu and Alimosho LGAs in Lagos state, and AMAC and Bwari LGAs in FCT.

#### Phase 2: selection of Photovoice study participants

From the cohort of 639 WRA who participated in the descriptive and cross-sectional prevalence survey, 24 women were selected by stratified purposive sampling. Women were selected from the four LGAs across both states. Based on the significantly higher population, more women were selected from the two LGAs in Lagos than in FCT. Of the 24 women selected, seven each were from Ikorodu and Alimosho LGAs in Lagos state with six from AMAC LGA and four from Bwari LGA in FCT. Across each LGA, the selected women were categorized into women living with or at risk of NCDs. The PhotovoicePhotovoice group participants (n = 24) were all WRA ranging from ages 22–48 years of age. These women represented four distinct urban and peri-urban/rural areas in Nigeria: Alimosho, AMAC, Bwari, and Ikorodu LGAs. (see Table 1).

**Table 1:** Demographic information of the study participants

Participants’ characteristics	Lagos		FCT		Total	
	Number	Percent	Number	Percent	Number	Percent
*Age range (in years)*						
20–29	2	14.3	3	30.0	5	20.8
30–39	5	35.7	5	50.0	10	41.6
40–49	7	50	2	20.0	9	37.5
*Level of education*						
Primary School completed	2	14.3	0	0.0	2	8.3
Secondary School Completed	7	50	6	60.0	13	54.2
College or University completed	5	35.7	4	40.0	9	37.5
*Marital status*						
Single	1	7.1	1	10.0	2	8.3
Married	13	92.9	9	90.0	22	91.7
*Employment status*						
Formal employment	4	28.6	1	10.0	5	20.8
Self employed	6	42.9	7	70.0	13	54.2
Unemployed	3	21.4	2	20.0	5	20.8
Student	1	7.1	0	0.0	1	4.2
*Presence of chronic disease/risk factor of a chronic disease*						
Hypertension only	5	35.7	4	40.0	9	37.5
Diabetes only	1	7.1	0	0.0	1	4.2
Diabetes & hypertension	4	28.6	0	0.0	4	16.7
At least one risk factor for chronic disease (e.g. overweight, truncal obesity, low physical activity, low vegetable consumption, smoking and alcohol intake)	4	28.6	6	60.0	10	41.6

#### Phase 3: orientation, consent and training workshops

An introductory workshop was held with the WRA and the research team in each state. The research team comprised clinical and public health practitioners (from mDoc Healthcare) who educated women on the background, purpose, and methods of the PhotovoicePhotovoice study. Consent was obtained from all participants, and they were subsequently trained on how to obtain informed consent from other subjects or owners of the property featured in the photos.

Each woman was provided with a digital camera (Lyyes Digital Camera, 2.7" Mini Camera HD 720P Digital Point Shoot Camera 8X Zoom) and trained to capture photos that conveyed their current health, healthcare utilization and engagement, and daily experiences that influence their health.

During the workshop, the women discussed their lives and their difficulties with accessing care, medications, healthy and nutritious foods, and hardships at home and how they believe these components impact their ability to prevent and manage chronic diseases The research team then spent time demonstrating to the women how to use the camera in a stepwise practical session. Based on initial conversations about their daily experiences and the impact on their health, women were asked to capture their experiences in photos over 6 days using the cameras.

#### Phase 4: data collection and developing stories from photos

After 6 days, the research team held one-on-one follow-up semi-structured interviews with each woman to discuss and review the photos. Each woman sat with a mDoc Healthcare research team member and described what each photo depicted. In some instances, multiple photos were taken of the same activity or experience in one or more locations. For example, a participant might have taken ten photographs at the market. The research team member would have the woman describe what was happening while at the market and also in each specific photograph at the market. From the photos, discussions uncovered challenges related to their lives and community. A total of 1680 photos were captured by the women.

These interviews were semi-structured with questions that included the following: (1) “What was happening in the photo?”; (2) “How does this photograph relate to your life?”; (3) “Why do you believe this problem exists?”; and (4) “How do you cope with the obstacles depicted in the photograph?”. These discussions were audio-recorded using the voice memo application on a mobile device and later transcribed. When necessary, follow-up calls were made to obtain more clarity around the occurrences of events in the photographs.

The photos and audio recordings from each woman were filed in different named folders on Google Drive. An affinity diagram was used to develop themes that categorized the barriers depicted in the photographs. Accompanying captions were developed for each photograph selected for each thematic area. Copies of each woman’s photographs were made available to her.

#### Ethical consideration

This study was approved by the National Health Research Ethics Committee of Nigeria and the Institutional Review Board of School of Public Health, Johns Hopkins University, Baltimore (IRB No. 00009452). Written consent to participate and share photos were obtained from the women at the beginning of the study. The women reviewed and signed consent and photo release forms with a research team member and completed a demographic questionnaire.

## Results

Although 58.3% of the women selected had a chronic disease (Hypertension 37.5%, Diabetes 8.3%, Hypertension, and Diabetes 12.5%), women did not have to have a diagnosis of chronic disease to participate in the study as it was important to learn factors placing women at risk of chronic diseases as well.

Through data analysis of 24 one-on-one interviews, we identified 6 overarching themes that depicted the barriers experienced by women:

1. *Food, and nutrition*
2. *Home and family,*
3. *Neighborhood-built environment,*
4. *Economic instability,*
5. *Religion and spirituality and*
6. *Low prioritization of self-care.*

### Food and nutrition

**Fig. 2:**
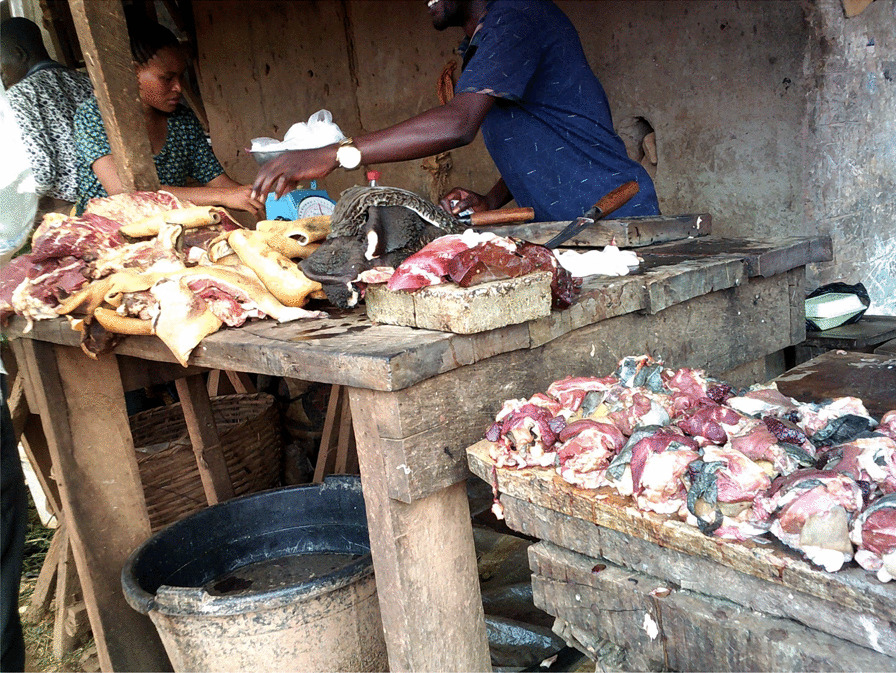
A market stand where meat is butchered and sold

Many women noted that they struggled with maintaining a nutritious diet. They consumed street food and prioritized carbohydrate-rich meals, such as rice, cassava grain (‘eba’), and yams frequently. The majority of the women’s diets consisted of more red meat than white meat (Fig. 2). The women also noted that they frequently prioritized their children over themselves when purchasing and preparing meals by saving the more nutritious food items (e.g., chicken) for their children and eating less nutritious foods themselves (e.g., red meat). Only when funds permit or during religious fasts did the women purchase fruits and vegetables as they recognize their nutritious value. Women living with diabetes noted the difficulty of having to eat different food items from their family members.

“I cannot eat the same food as everyone else in the house. I have to be more mindful of the food I eat because of my diabetic condition. I am eating pap with beans while the others are eating rice and moi moi.”—(IP 1) Ikorodu LGA, Lagos.

“This is the stand in the market where I buy my meat. I only eat red meat but I buy chicken for my children.”—(IP 2) AMAC LGA, FCT.

**Fig. 3:**
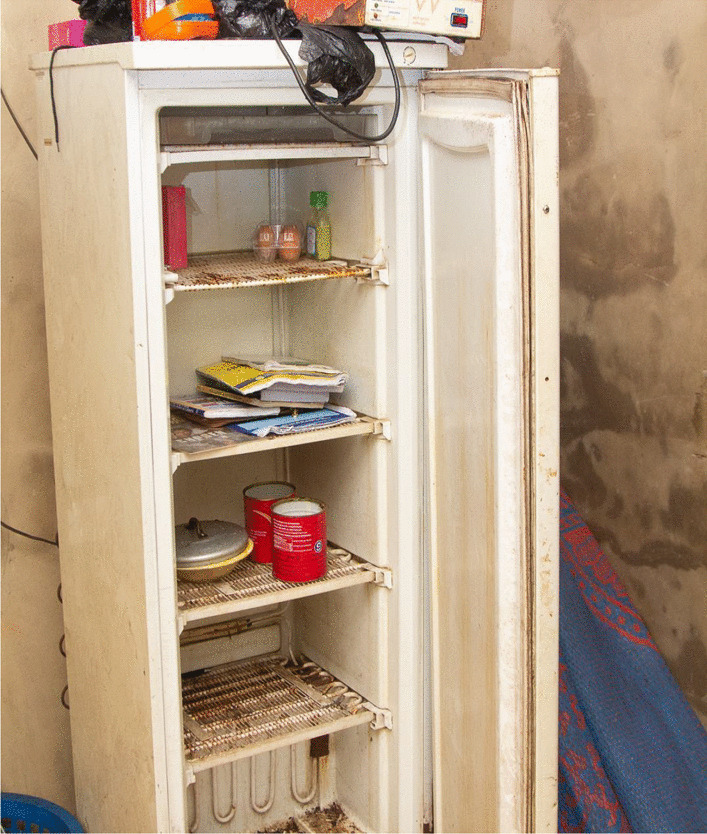
A fridge, without any power supply, now serving as a cabinet for storing items

“My fridge is used as a cabinet for storing household items. We buy food and cook once I make any money that day. I have no single raw food at home right now.” (Fig. 3)—(IP 3) Alimosho LGA, Lagos.

### Home and family

The women agreed that home and family had a salient role in influencing how they managed their health. They noted that high demands are often placed on women at home, stating that even if they had to work, they were still expected to be the primary caregiver for their children and cook and clean for the home. The women often had to prioritize their household, family responsibilities, and community involvement over their health.

“I’m a wife, a mother of four children (aged 13–24 years), and I run two small businesses. I also take care of the home, serve in the church and manage my health condition (hypertension). My life is very busy.”—(IP 3) Alimosho LGA, Lagos.

Despite this, the women appreciated spending time with their families as it added joy to their lives. Parents' views also had a strong influence on the women. One of the women interviewed was the daughter of a traditional healer. She had never seen a doctor before because her mother usually tended to all of her illnesses with herbs. Another woman made it her priority to save funds towards regular doctor visits after witnessing her parents struggle with health ailments.

**Fig. 4:**
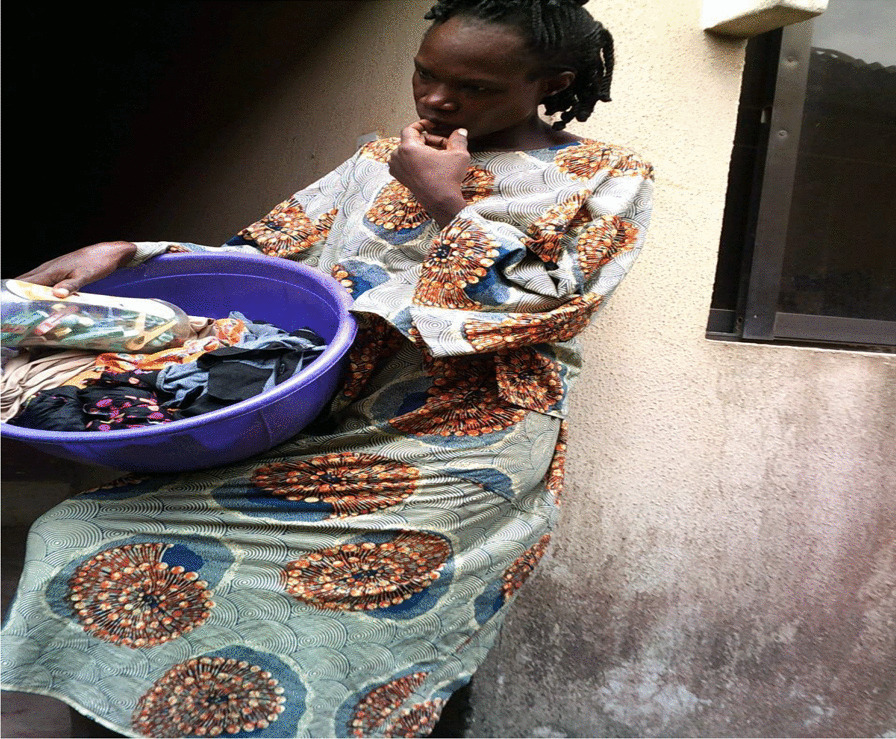
(IP 4) washing clothes

“This is pregnant me washing clothes on a Saturday morning. I was tired from the week. Working, shopping for the house, cooking, cleaning, taking care of the children and my husband was tiring. But I have no option but to wake up and do the washing.” (Figure 4)—(IP 4) Ikorodu, LGA Lagos

### Neighborhood built environment

The women also described the impact of poor roads and traffic on their health. While they understood that regular physical activity is key for people with chronic disease, they reported limited physical activity due to obstructed and flooded roads, hazardous litter, lack of sidewalks and heavy traffic.

“Bad government, bad road. When you want to pass the road you have to fold your trousers to get through. If rain falls it’s even worse. Sometimes people just leave their cars because it breaks down on the road.”—(IP 6) Bwari LGA, FCT.

**Fig. 5:**
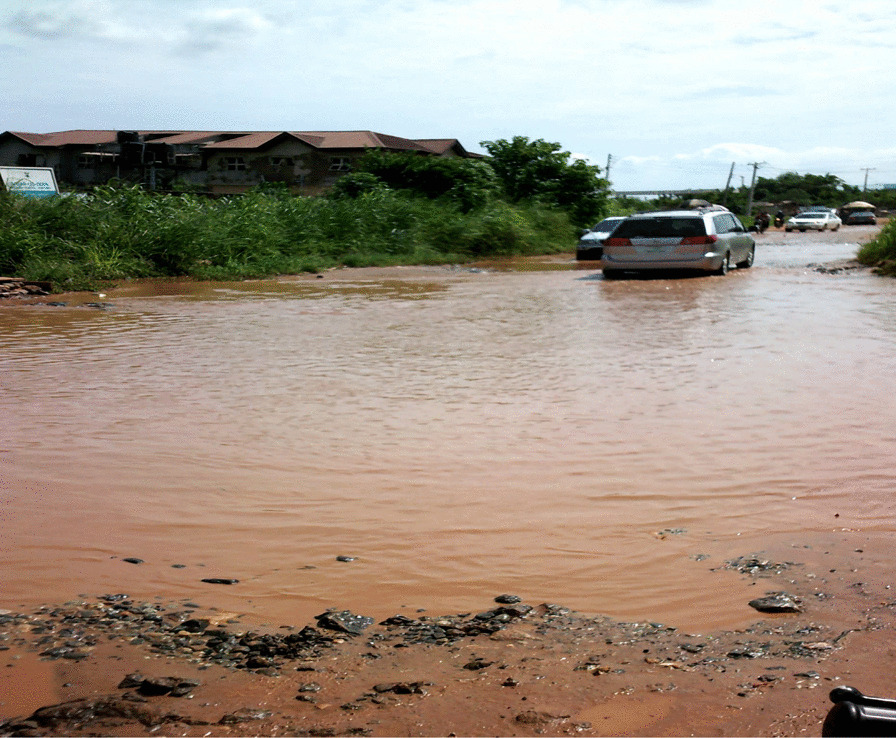
A road in FCT, flooded due to the rain

The women noted that the frequent flooding of unpaved roads led to breeding grounds for mosquitoes (Fig. 5). This increased the risk of malaria and added financial strain for the women’s families. Flooded roads also negatively impacted the access for patrons to the women’s businesses. This reduced foot traffic decreased the incomes for already struggling families who then did not have the disposable income to manage their health. The heavy traffic caused by flooded roads also dissuaded women from visiting clinics and hospitals. The time spent in traffic also made the women more likely to purchase unhealthy street food, experience reduced family time, and endure increased stress levels.

**Fig. 6:**
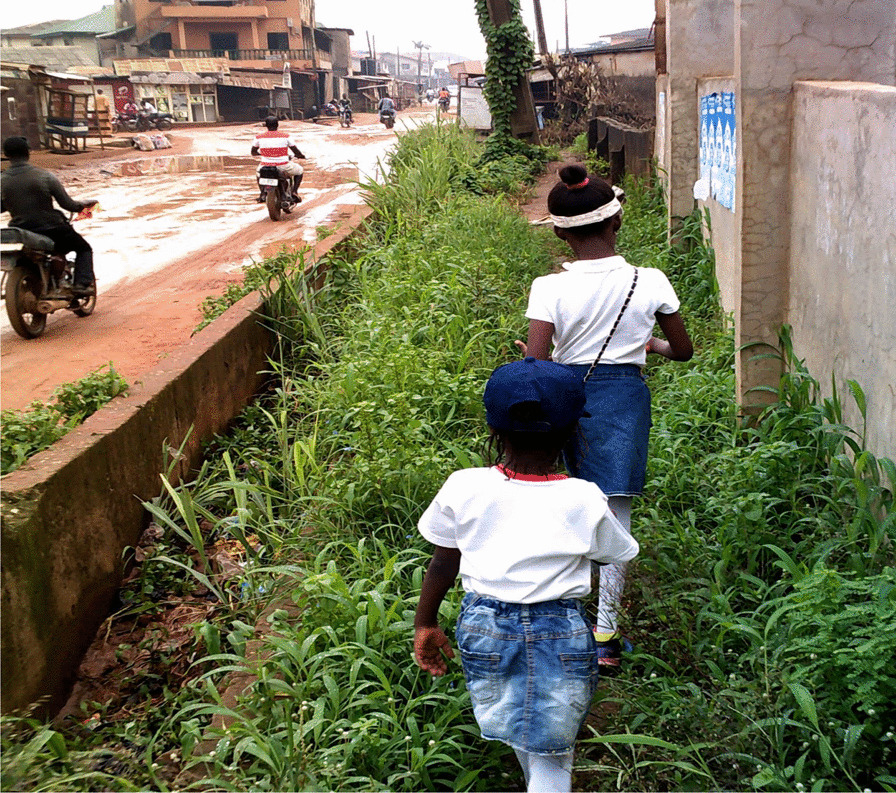
(IP 4)’s daughters walking in a grassy sidewalk

“My daughters and I are walking to our church which is about a 5-minute walk. We had to walk in the bush to avoid the water on the road.” (Figure 6)—(IP 4), Ikorodu LGA, Lagos

“I use the pedestrian bridge every time I cross the road. It’s a form of exercise for me. There used to be no light on the bridge at night and they would steal people’s bags. But the government recently installed solar panel lights so now it’s well-lit and safer to cross at night.”—(IP 7) AMAC LGA, FCT.

### Economic instability

**Fig. 7:**
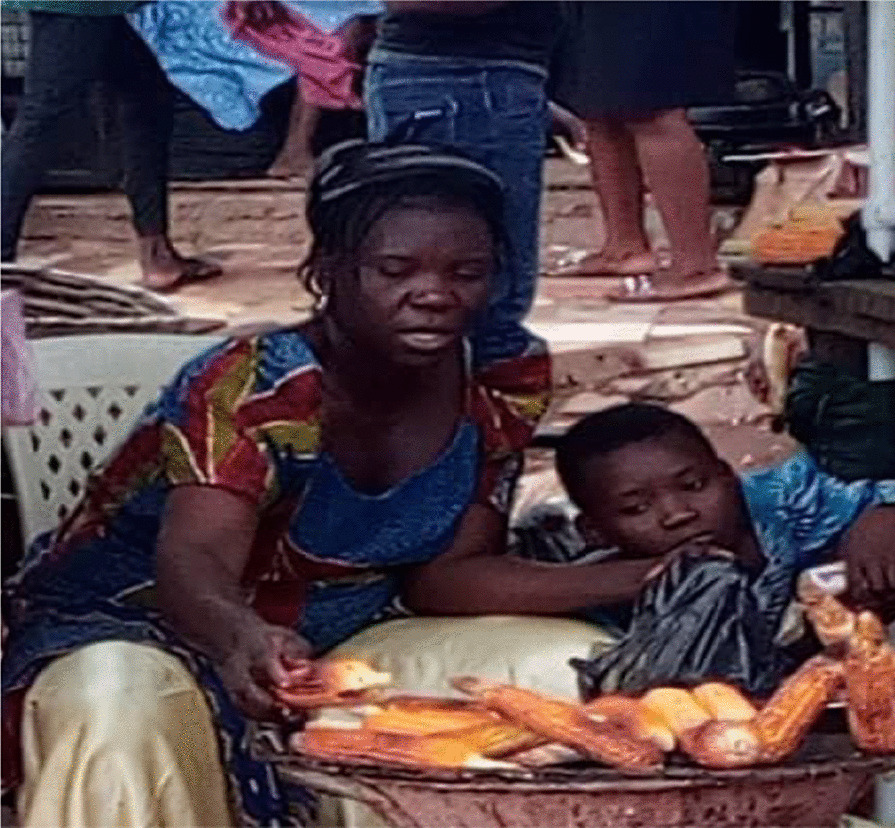
(IP 6) roasting corn for sale by the road

The women who participated in the study were employed in various industries, some owned shops and businesses, worked as food sellers or traders at local markers or had office jobs in Lagos or FCT (Fig. 7). Most of them were self-employed with a combined household monthly income of N0- N75,000 ($0–208 USD). Majority of the women also lacked health insurance and indicated they did not have the disposable income for out-of-pocket expenditures for health. The women saw their work as a source of pride but also as a necessity to support their families. For women living with diabetes, having to follow a different diet from that of their family added financial strain on resources and convenience. In addition, lower-income women noted that they had to deal with frequent power outages and lack of running water. This added to their stress and limited the time and attention they could spend on their health.

“To make more money I also have a second business selling kerosene in recycled plastic bottles”—(IP 8) Alimosho LGA, Lagos.

Meals are uncertain in low-income families who often are unable to afford to stock up enough food at home. Depending on the income made in a day, women frequently bought just enough food for their families to eat on that day. Several women engaged in subsistence (urban) farming to grow their vegetables and also participated in fish farming.

“I am a mother of five children, and I roast maize by the road to support my family. From this business, I save N200 ($0.55) every week so I can visit the doctor at least once every three months.”—(IP 6) Bwari LGA, FCT.

**Fig. 8:**
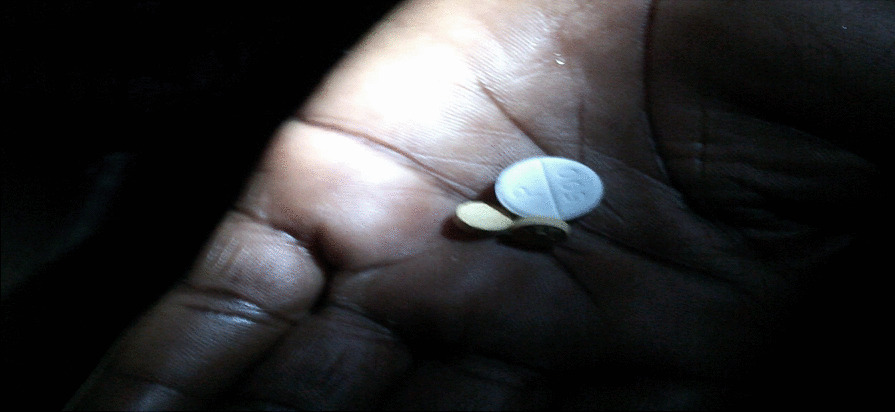
(IP 5) viewing her medication under a flashlight

“At this time there was no electricity in the house because they took light. I needed to take my medication for my hypertension. I had to use a flashlight to get around and see what I’m doing.” (Figure 8)—(IP 5) Alimosho LGA, Lagos.

“In addition to the farm we recently started behind my house, we also decided to get fishponds. So, we sell food from the farm and the fish. With the way the economy is, I need to feed my family and start making more money which is why we’re doing this.”—(IP 2) AMAC LGA, FCT.

### Religion and spirituality

**Fig. 9:**
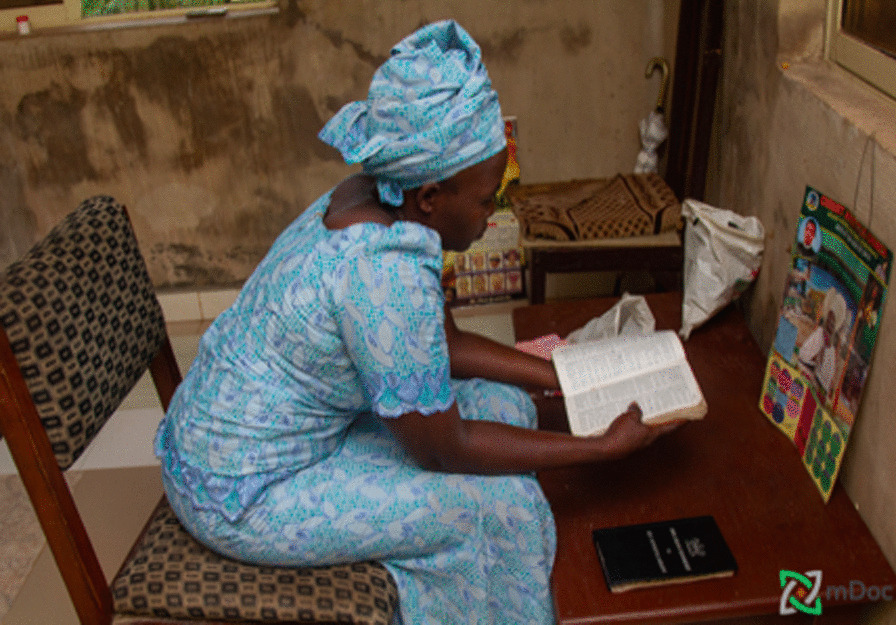
(IP 13) praying at home

Religion played a significant role in the daily lives of women and how they approach their health. Women’s spirituality, including traditional worship and beliefs, was a major part of the women’s identities, and they made sure to dedicate time weekly to their religion (Fig. 9). Many women were deeply involved in their churches and mosques. Religion influenced women’s perceptions of life and how they dealt with the many daily challenges they face.

“I have a strong relationship with God. God is a source of peace when I am stressed about finances and worried about not having money to buy my drugs. When my blood pressure is high (often due to no money to buy drugs), I commit it to God in prayer.”—(IP 3) Alimosho LGA, Lagos.

Women also turned to religion to address their chronic illnesses and as a cure all when dealing with stress and depression.

“This is me coming out of the church. I pray about my health every Sunday as I am diabetic.”—(IP 13) Ikorodu LGA, Lagos.

### Under prioritization of self-care

While health was a major concern for the women, many felt that they were incapable of managing it adequately. The women with limited funds found themselves having to prioritize food and other household costs over the costs of their healthcare and medication. The women usually only bought medication to treat their chronic conditions if there were available funds. The women noted that they often had to purchase their medication “one sachet at a time”, which would only last for a couple of days as they could not afford to buy in bulk. Not being able to afford medication made it challenging to manage their chronic illness and women with hypertension would frequently experience monthly episodes of elevated blood pressure readings. Many women viewed going to hospitals as expensive and time-consuming. They often experienced long waiting times (up to three hours), which made many avoid visiting health facilities. Many women chose to self-medicate at local pharmacies because it was easier and more affordable.

“I had to leave work to go to the hospital because I was having malaria symptoms. Most times I prefer to self-medicate because of the long wait times at the hospital. Sometimes you have to wait 2–3 days to get test results. The probability that you will get the results when you need it is low because there are so many people that go to Wuse General Hospital.”—(IP 9) AMAC LGA, FCT.

“I go to a lab and pharmacy to get checked up and get drugs because the hospital in my area is expensive and the government clinic near my house is never open. When I’m sick I will go to a hospital.”—(IP 10) Bwari LGA, FCT.

**Fig. 10:**
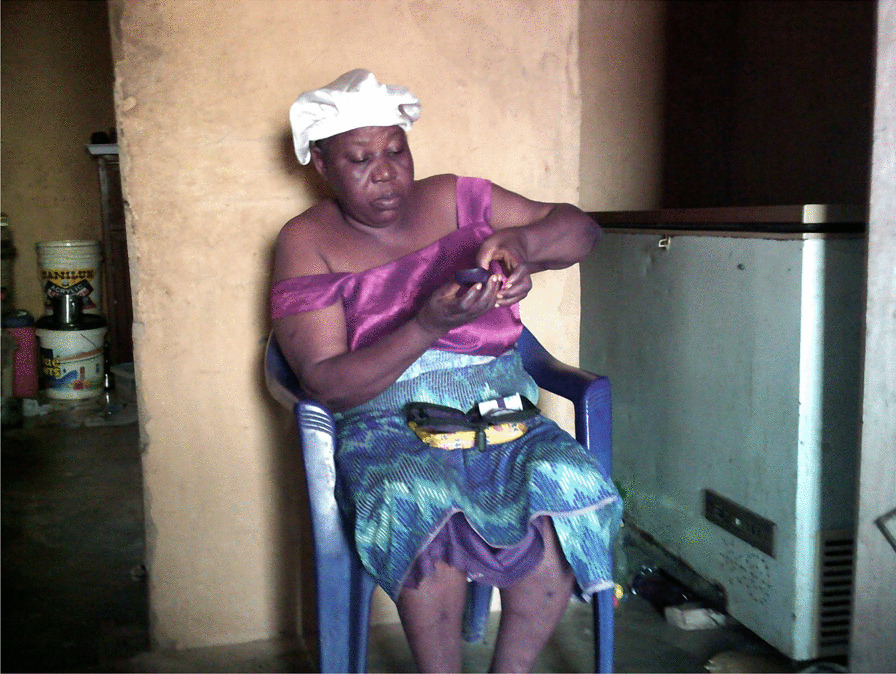
(IP 1) testing her blood sugar levels

“This is me testing my blood sugar. I am diabetic so I have to always check my sugar levels. It’s easy for me to check my sugar level at home by myself. It is only tough when I run out of strips because I have to look for money to buy more.” (Figure 10)—(IP 1) Ikorodu LGA, Lagos

## Discussion

This study presents an opportunity to explore the barriers to health that WRA living in urban and peri-urban/rural areas in Nigeria encounter and how they impede positive maternal health outcomes. Through this approach, community members’ and women’s voices can play a major role in policy and systemic change [15].

The women in our study were not naive about their noncommunicable disease status or risk factors. They did not have access to an enabling ecosystem to help them manage their health. There were multiple systemic problems identified in this study that hinder safe and efficient healthcare management in WRA in Nigeria.

Nigeria continues to struggle with poor infrastructure, which negatively impacts the health of all its citizens but especially poor women living with chronic disease who are particularly vulnerable to the challenges of intersecting inequalities. Research has suggested that walking is the most common and feasible form of physical activity, but the absence of adequate infrastructure to support walking can make physical activity impossible for many women [16]. According to the Global Observatory for Physical Activity, the prevalence of physical inactivity in Nigerian women was 26% [17]. As conveyed in this study, the hazards of walking on the roads due to constant traffic, poorly maintained roads, waste dumping, and subsequent flooding of roads prevented women from participating in physical activities in their communities. Regulations should be instituted to create more enabling infrastructure to promote health.

In addition, the inefficient public transportation system can make traveling to and from healthcare facilities challenging and time-consuming thereby impeding access to care. Investments in reproductive and maternal care must consider structural investments that address the accessibility factors to health service utilization in vulnerable populations.

Low health insurance coverage coupled with the cost of healthcare services lead to the poor spending a disproportionately higher amount of disposable household income on healthcare [18]. This deters women from seeking preventive and promotive services which could reduce the burden of chronic illnesses. As this study shows, women frequently under-prioritize their health. Women consistently chose their families over their own health. The interconnected nature of gender and class has created an inequitable environment that continuously places women in compromising situations when it comes to their health. Many of the women in our study were self-employed and their daily meals depended on their daily income. Managing chronic illnesses can have devastating economic effects on households, so it is not a surprise that many women in our study overlooked prioritizing funds for their care. In the setting of limited finances, and relatively high out-of-pocket expenditures, women of reproductive age living with or at risk for NCDs would benefit from community health insurance and community-based health models to increase access to healthcare and supplement household spending. Policies in the country should focus on addressing the structural and economic barriers that prevent women from living a happier, healthier, and more productive life.

This Photovoice study empowered a group of WRA to document aspects of their daily lives that impact their ability to prioritize their health. Employing interventions to empower and engage communities is key to strengthening the Nigerian healthcare system. It is important to highlight these barriers to strategically address the rising prevalence of chronic conditions amongst WRA and to provide data that can support the drafting of policies that enhance women’s agency and prioritize interventions that create the enabling ecosystem for lasting change.

### Strengths and limitations

One strength of this study was the inclusion of both pregnant and non-pregnant women for a broader perspective. Another strength is the use of Photovoice methodology to bring the voices of WRA to the forefront, given that their voices are often not reflected in research.

Limitations of the study included that the women who participated were from only LGAs in the South West and North Central geopolitical zones of Nigeria with a lack of representation from the other four geopolitical zones. There were also no participants from fragile and conflict affected zones. While the barriers elicited by the Photovoice study are those that many women in Nigeria experience, there may be certain barriers not seen in the study that would only affect women in fragile and conflict affected zones.

## Conclusion

The barriers highlighted in this Photovoice study show that addressing the indirect causes of maternal mortality in Nigeria is a multi-dimensional challenge that requires public and private sector level involvement. Policies and interventions should be women- and community-centred. The enabling environment must be built to address the intersectional inequalities that women face that predispose them to poor health especially at the time of pregnancy. In promoting reproductive and maternal health, it is paramount to identify and address the risk factors in societal constructs that jeopardize positive maternal mortality and morbidity outcomes in women.

## Data Availability

The data that support the findings of this study are available on request from the corresponding author AO. The data are not publicly available due to them containing information that could compromise the privacy of the participants of the study.

## Abbreviations

CBPR: Community based participatory research
FCT: Federal capital territory
GDM: Gestational diabetes mellitus
HPD: Hypertension pregnancy disorders
LGA: Local government area
MMR: Maternal mortality ratio
NCD: Non-communicable disease
WRA: Women of reproductive age
